# AESIS-1, a Rheumatoid Arthritis Therapeutic Peptide, Accelerates Wound Healing by Promoting Fibroblast Migration in a CXCR2-Dependent Manner

**DOI:** 10.3390/ijms25073937

**Published:** 2024-04-01

**Authors:** Seung Beom Park, Yoolhee Yang, Sa Ik Bang, Tae Sung Kim, Daeho Cho

**Affiliations:** 1 Department of Life Sciences, College of Life Sciences and Biotechnology, Korea University, Anam-dong 5-ga, Seongbuk-gu, Seoul 02841, Republic of Korea; kaolu84@korea.ac.kr; 2 Kine Sciences, 6F, 24, Eonju-ro85gil, Gangnam-gu, Seoul 06221, Republic of Korea; yhyang@kinesciences.com (Y.Y.); cdhkor@korea.ac.kr (D.C.); 3 Department of Plastic Surgery, Samsung Medical Center, School of Medicine, Sungkyunkwan University, Gangnam-gu, Seoul 06351, Republic of Korea; si55.bang@samsung.com; 4 Institute of Convergence Science, Korea University, Anam-dong 5-ga, Seongbuk-gu, Seoul 02841, Republic of Korea

**Keywords:** wound healing, AESIS-1, peptide, fibroblast, migration, CXCR2, ERK, p38

## Abstract

In patients with autoimmune disorders such as rheumatoid arthritis (RA), delayed wound healing is often observed. Timely and effective wound healing is a crucial determinant of a patient’s quality of life, and novel materials for skin wound repair, such as bioactive peptides, are continuously being studied and developed. One such bioactive peptide, AESIS-1, has been studied for its well-established anti-rheumatoid arthritis properties. In this study, we attempted to use the anti-RA material AESIS-1 as a therapeutic wound-healing agent based on disease-modifying antirheumatic drugs (DMARDs), which can help restore prompt wound healing. The efficacy of AESIS-1 in wound healing was assessed using a full-thickness excision model in diabetic mice; this is a well-established model for studying chronic wound repair. Initial observations revealed that mice treated with AESIS-1 exhibited significantly advanced wound repair compared with the control group. In vitro studies revealed that AESIS-1 increased the migration activity of human dermal fibroblasts (HDFs) without affecting proliferative activity. Moreover, increased HDF cell migration is mediated by upregulating chemokine receptor expression, such as that of CXC chemokine receptor 2 (CXCR2). The upregulation of CXCR2 through AESIS-1 treatment enhanced the chemotactic reactivity to CXCR2 ligands, including CXC motif ligand 8 (CXCL8). AESIS-1 directly activates the ERK and p38 mitogen-activated protein kinase (MAPK) signaling cascades, which regulate the migration and expression of CXCR2 in fibroblasts. Our results suggest that the AESIS-1 peptide is a strong wound-healing substance that increases the movement of fibroblasts and the expression of CXCR2 by turning on the ERK and p38 MAPK signaling cascades.

## 1. Introduction

In previous clinical studies, patients with autoimmune disorders, including rheumatoid arthritis, have displayed delayed wound healing [1,2,3]. Patients with rheumatoid arthritis (RA) display an increased incidence of wounds, including leg and foot ulcers [2], and these wounds can take months or even years to heal. Such delayed- or slow-healing wounds are a common phenomenon in these patients [3]. Moreover, a recent study reported that patients who take disease-modifying antirheumatic drugs (DMARDs) show significantly shorter wound-healing times [4].

Wound healing is a finely orchestrated biological process involving the collaborative efforts of various cell types and molecular mediators [5,6,7,8]. Among the essential participants in this delicate symphony of regeneration, fibroblasts play a key part in wound healing, especially during the proliferative and remodeling phases [9,10]. Fibroblasts are known to have a function in the wound-healing process through (1) migration into the wound bed, (2) collagen synthesis, (3) extracellular matrix (ECM) formation, (4) wound contraction, (5) angiogenesis, and (6) the restoration of tissue integrity [11,12]. Immediate fibroblast migration to the wound bed is an important wound-healing process that can initiate various fibroblast functions [10,11,13]. Numerous chemotactic components, including chemokines, growth factors, cytokines, and ECM, are known to be involved in fibroblast migration [14,15,16]. Among these components, the interplay between chemokine receptors, including CXC chemokine receptor 2 (CXCR2), and their counterpart chemokines is an important factor in regulating wound-healing outcomes [17,18]. Previous studies report that CXCR2-p38 Mitogen-activated protein kinase (MAPK) signaling cascades are significantly involved in the migration process [19,20]. MAPKs are a class of serine/threonine protein kinases that are essential for signal transduction pathways, which play a role in cell migration, survival, differentiation, and proliferation [21,22]. MAPK signaling cascades were also found to be involved in fibroblast activation, proliferation, and migration [14,22,23,24].

Bioactive peptides are a group of biological substances composed of amino acids and are known to have beneficial activities in human health [25]. According to its diverse biological activities, such as antimicrobial, antithrombotic, antihypertensive, opioid, immunomodulatory, mineral-binding, and antioxidative, it is continuously highlighted in the pharmaceutical industry [26,27]. Especially, a group of peptides with antimicrobial activity, including human endogenous β-defensins (hBDs) 1–3, cathelicidin antimicrobial peptide (LL-37), and dermcidins, is known to have a beneficial role in wound-healing processes [28,29]. Recent studies have shown that computational methods can also predict other peptides or proteins with antimicrobial properties that are significantly involved in wound-healing applications [30,31,32].

A previous study of the novel 19-amino-acid bioactive peptide AESIS-1 found it to have therapeutic activity for rheumatoid arthritis treatment [33]. Based on several reports of delayed wound-healing side effects common in rheumatoid arthritis patients [1,3] and reports that rheumatoid arthritis therapeutics can accelerate the wound-healing process [4], we conducted a study on the possibility of applying AESIS-1, an anti-RA agent, as a wound-repair therapeutic. In this study, we found that the AESIS-1 peptide significantly improved wound repair in vivo in a chronic wound-healing model in diabetic mice. AESIS-1 was found to increase the migration activity of human dermal fibroblasts by upregulating CXCR2 expression and activating the ERK and p38 MAPK signaling pathways.

## 2. Results

### 2.1. AESIS-1 Exerted Acceleration of Wound Healing In Vivo

**Figure 1:**
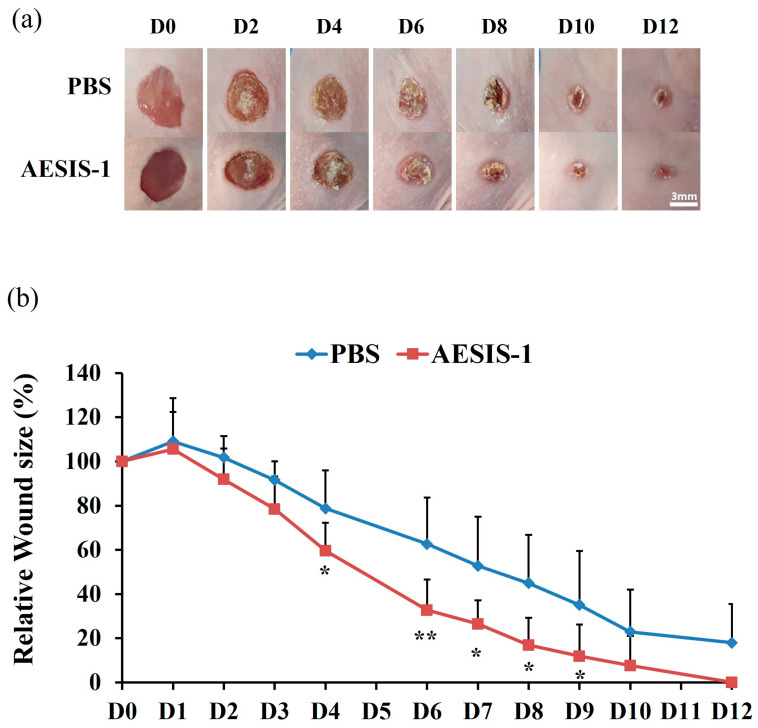
AESIS-1 treatment-accelerated wound healing in an in vivo model. Full-thickness wounds were created on the backs of diabetic BALB/c-nude mice according to the protocol described in the Section 4. PBS control or AESIS-1 peptide (0.5 μg/wound) treatments were applied 5 times at 24 h intervals beginning on day 0. (**a**) A photographic record of wound healing was compiled from day 0 to day 12, once every two days. Scale bar 3 mm. (**b**) The relative sizes of the wounds were analyzed as a ratio of the measured wound size to the original injury size at 0 days. The experiments were performed on a total of 7 or 8 mice per group (*n* = 8 for the PBS-treated group, *n* = 7 for the AESIS-1-treated group). Error bars, mean ± SD. A non-paired and two-tailed Student’s *t*-test was used for statistical analysis. * *p* < 0.05, ** *p* < 0.01 (versus PBS control).

In previous research, the novel synthetic peptide AESIS-1 had preventive effects on a collagen-induced arthritis (CIA) model in mice, which is the most widely studied model of rheumatoid arthritis [33,34]. To investigate the efficacy of the AESIS-1 peptide on chronic wound repair, a diabetic wound-healing model was used in this study. The backs of diabetic BALB/c-nude mice were extracted using a 6 mm diameter round punch to generate a full-thickness wound. Then, a phosphate-buffered saline (PBS) control or AESIS-1 peptide was administrated to the acute wound site five times at 24 h intervals. Wound sizes were then monitored and analyzed over 12 days. The results show that, in mice, AESIS-1 treatment significantly accelerates wound healing compared with the PBS control group (Figure 1).

### 2.2. AESIS-1 Increases the Migration Activity of Fibroblasts without Affecting Fibroblast Proliferation

**Figure 2:**
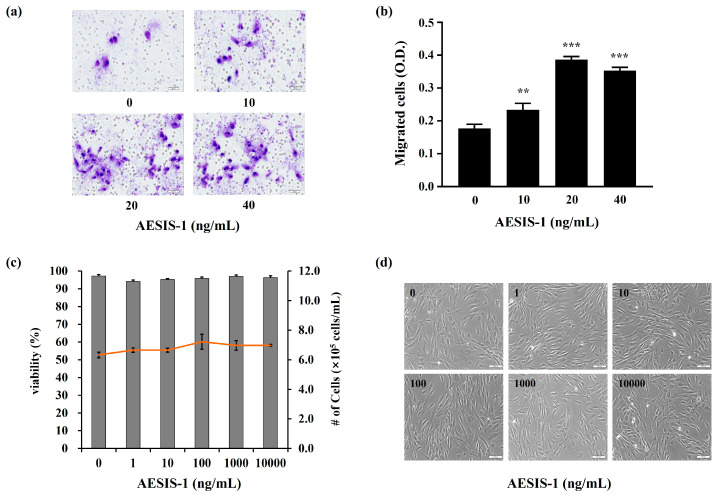
The AESIS-1 peptide enhances HDF cell migration. To investigate fibroblast migration, 2 μg/mL of mitomycin-C-treated HDF cells (7 × 10^4^ cells/well) was resuspended in serum-free media seeded in the upper chamber of the transwell and then incubated for 12 h. The lower chamber was treated with AESIS-1 at the indicated dose (0, 10, 20, 40 ng/mL) in 1% FBS media. After 12 h of incubation, the migrated cells in the lower compartment of the transwell inserts were fixed and stained with 0.5% crystal violet. This stain was then solubilized and extracted with 10% acetic acid, and the absorbance was read using a spectrophotometer at 570 nm. To investigate fibroblast proliferation, HDF cells were seeded in a 6-well plate, and a broad concentration range of AESIS-1 was applied for 24 h under serum-free conditions. After incubation, the viability of the cells and the number of cells were measured with a Trypan blue exclusion assay. (**a**) A photographic record of the membrane image of the lower compartments of transwell inserts. Scale bars 50 μm. (**b**) A bar graph representing the eluted density of migrated cells in each group. The absorbance was measured using a microplate reader at 570 nm. (**c**) A graph showing the measured cell number and calculated viability. (**d**) A microscopic image of the density and morphology of HDF cells. Scale bars 100 μm. Error bars in Figure 1b, c represent the mean ± SD from three independent experiments. One-way analysis of variance (ANOVA) with Turkey’s multiple comparisons test was used for statistical analysis. ** *p* < 0.01, *** *p* < 0.001 (versus without the AESIS-1-treated control).

Immediate cell movement of the fibroblast to the wound bed is known to be a very important process in wound healing, and the process is regulated by various mediators, including chemokines [11,12,16]. In the present study, we performed transwell migration assays to observe the changes in the migration activities of human dermal fibroblast (HDF) cells with AESIS-1 treatment. HDF cells were pre-treated with mitomycin C to prevent the proliferative effect. Figure 2a,b show that the migration of HDF cells was statistically significantly increased in all groups that were treated with AESIS-1. The most effective concentration for cell mobility was the 20 ng/mL AESIS-1 treatment condition, and this was used in subsequent experiments (Figure 2b).

After the migration of fibroblasts to the wound areas, it is known that they actively proliferate to heal wounds [11]. Therefore, we tested the effects of AESIS-1 on the proliferation and viability of HDF cells. Increased proliferative activity or decreased cell viability were not observed in this study over a broad range of AESIS-1 treatments (Figure 2c). Furthermore, no change in HDF cell morphology was detected due to AESIS-1 (Figure 2d).

### 2.3. Enhanced Fibroblast Migration by AESIS-1 Is Mediated by Upregulating CXCR2 Expression

**Figure 3:**
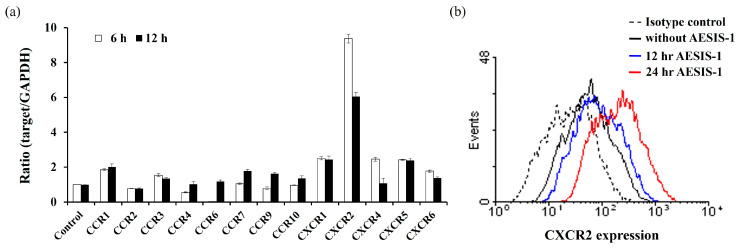
AESIS-1 enhances expression of chemokine receptors in HDF cells. (**a**) mRNA expression of human chemokine receptors (CCR1, CCR2, CCR3, CCR4, CCR6, CCR7, CCR9, CCR10, CXCR1, CXCR2, CXCR4, CXCR5, and CXCR6) was determined with real-time PCR of total RNA isolated from 20 ng/mL AESIS-1-treated HDF cells (7 × 10^5^ cells/100 mm dish) for 6 or 12 h. (**b**) Surface protein expression of CXCR2 by HDF cells treated with 20 ng/mL AESIS-1 for 12 or 24 h was measured with flow cytometric analysis.

Chemokines and their receptors on resident cells are important modulators of wound healing in human skin through their contribution to regulating cell migration, tissue remodeling, and angiogenesis [16,35]. CXC chemokines and their receptors, including CXCR2, have a significant biological role in cutaneous wound healing [17,18]. To investigate whether AESIS-1 treatment affects the expression of the chemokine receptors on fibroblasts, HDF cells were treated with AESIS-1 for 6 or 12 h. Quantitative PCR results revealed that AESIS-1 caused an increase in the expression of various chemokine receptors, the largest of which was CXCR2 (Figure 3a). We also used protein levels to confirm a significant increase in the expression of CXCR2 with 12 or 24 h AESIS-1 treatments (Figure 3b).

**Figure 4:**
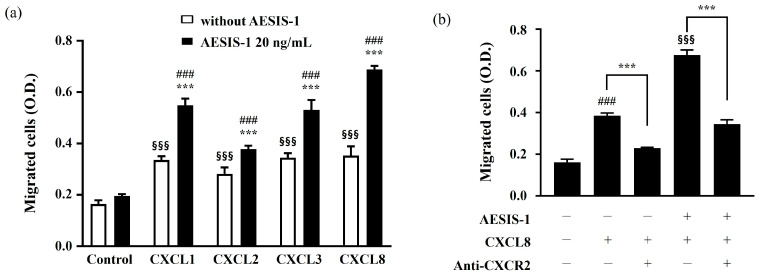
AESIS-1 enhances the migration of fibroblasts in a CXCR2-dependent manner. (**a**) HDF cells (1.5 × 10^6^ cells/150 mm dish) were treated with 0 or 20 ng/mL AESIS-1 for 24 h. The cells treated with 2 μg/mL of mitomycin C were seeded in the upper chamber of the transwell, then incubated for 12 h. The lower chamber was treated with 50 ng/mL of human chemokines (CXCL1, CXCL2, CXCL3, and CXCL8) in 1% FBS media. The bar graph represents the eluted density of migrated cells in each group. Error bars, mean ± SD. Two-way ANOVA with Dunnett’s multiple comparisons test was used for statistical analysis. *** *p* < 0.001 (versus the 20 ng/mL AESIS-1-treated control); §§§ *p* < 0.001 (versus without the AESIS-1-treated control); ### *p* < 0.001 (without the AESIS-1-treated control versus 20 ng/mL of the AESIS-1-treated group in each condition). (**b**) HDF cells (1.5 × 10^6^ cells/150 mm dish) were pre-treated with 4 μg/mL of anti-CXCR2 neutralizing antibody for 1 h, and then 20 ng/mL AESIS-1 was applied for 24 h. The cells harvested with 2 μg/mL of mitomycin C were seeded in the upper chamber of the transwell and then incubated for 12 h. The lower chamber was treated with 50 ng/mL CXCL8 recombinant protein in 1% FBS media. One-way ANOVA with Turkey’s multiple comparisons test was used for statistical analysis. ### *p* < 0.001 (versus without the AESIS-1-treated, CXCL8-treated, and anti-CXCR2-treated groups); §§§ *p* < 0.001 (versus without the AESIS-1-treated group, with the CXCL8-treated group, and without the anti-CXCR2-treated group); *** *p* < 0.001 (a comparison between treatments with and without anti-CXCR2 is presented).

Next, we investigated the effects of increased AESIS-1 migration by applying CXC chemokines such as CXC motif chemokine ligand 1 (CXCL1), CXC motif chemokine ligand 2 (CXCL2), CXC motif chemokine ligand 3 (CXCL3), and CXC motif chemokine ligand 8 (CXCL8), which are known to promote cell migration through their common chemokine receptor, CXCR2. The transwell migration results show that AESIS-1 treatment increased chemotactic reactivities against CXCL1, CXCL2, CXCL3, and CXCL8. Of the applied chemokines, the highest chemotactic responsiveness of HDF cells was observed against the CXCL8 chemokine (Figure 4a).

Fibroblast cell movement was measured after treatment with anti-CXCR2 neutralizing antibodies to verify whether the increased chemotactic activity was mediated by CXCR2. The results showed that AESIS-1 increases the migration of HDF cells and that this is linked to higher levels of CXCR2 (Figure 4b).

### 2.4. ERK and p38 MAPK Signaling Cascades Are Involved in AESIS-1-Enhanced Cell Migration and CXCR2 Expression

**Figure 5:**
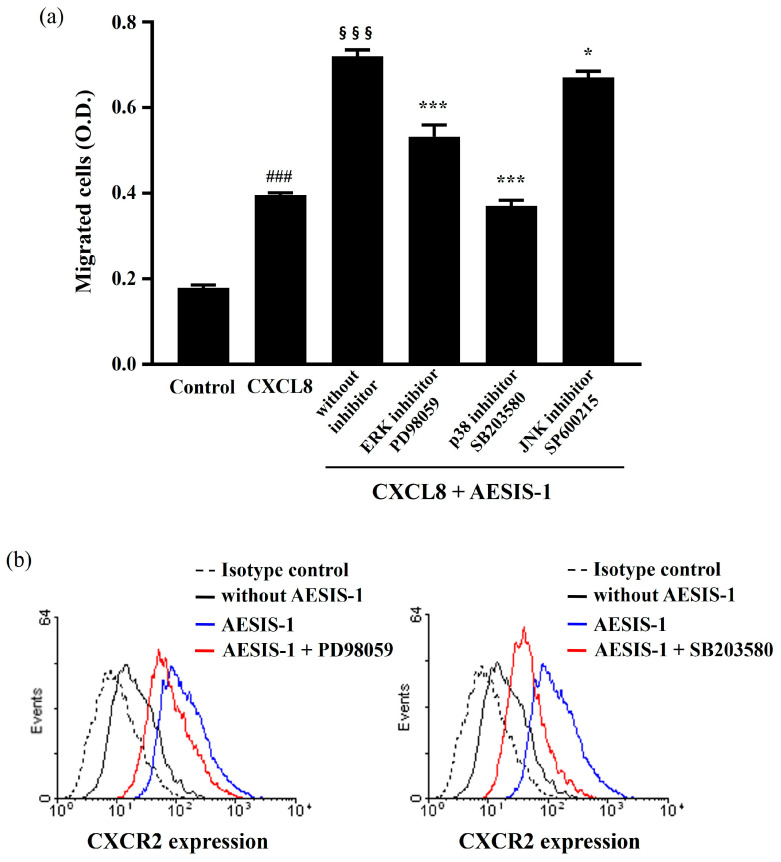
AESIS-1 enhances migration activities and CXCR2 expression through ERK and p38 MAPK signaling in HDF cells. Cells were treated with 20 ng/mL AESIS-1 for 24 h. MAPK inhibitors (ERK-PD98059, 10 μM; p38-SB203580, 10 μM; and JNK-SP600215, 20 μM) were added to cells 1 h before peptide treatment. (**a**) Transwell migration assay was performed using MAPK inhibitors and AESIS-1-treated HDF cells for 12 incubation hours with or without CXCL8 in lower chamber. Error bars, mean ± SD. One-way ANOVA with Turkey’s multiple comparisons test was used for statistical analysis. * *p* < 0.05, *** *p* < 0.001 (versus AESIS-1-treated group, with CXCL8-treated control); §§§ *p* < 0.001 (versus without AESIS-1-treated group, with CXCL8-treated group); ### *p* < 0.001 (versus without AESIS-1-, CXCL8-treated groups). (**b**) Surface protein expression of CXCR2 was measured with flow cytometric analysis after HDF cells were treated with AESIS-1 for 24 h with or without MAPK inhibitor pre-treatment.

According to previous results, AESIS-1 enhances the migration and expression of CXCR2 in HDF cells (Figure 2 and Figure 3). To identify the signaling cascades that regulate these consequences of AESIS-1 treatment, MAPK signaling was observed in this study. AESIS-1’s increase in chemotactic activities against CXCL8 was significantly decreased by blocking ERK and p38 MAPK with specific inhibitors (an ERK inhibitor, PD98059, and a p38 inhibitor, SB203580). Additionally, the JNK inhibitor SP600215 partially reduced the migration activities of AESIS-1 (Figure 5a). Moreover, the inhibition of ERK and p38 MAPK induced the downregulation of CXCR2 expression in the flow cytometric analysis (Figure 5b).

**Figure 6:**
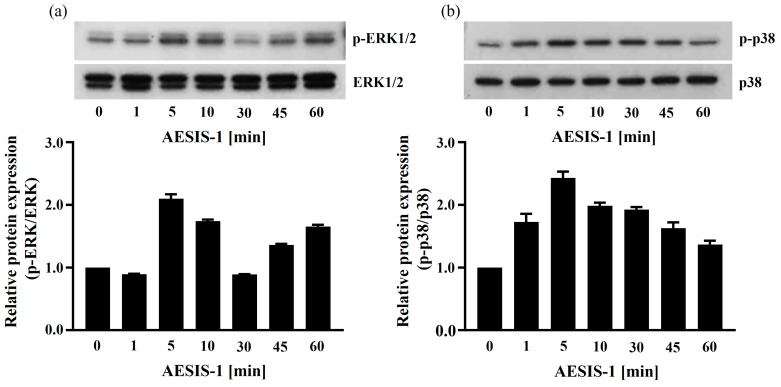
AESIS-1 activates the ERK and p38 MAPK signaling cascades in HDF cells. Cells (3 × 10^5^ cells/60 mm dish) in serum-free media were treated with 20 ng/mL AESIS-1 for the indicated durations (0, 1, 5, 10, 30, 45, and 60 min). Harvested cell lysates were analyzed with Western blotting as described in the Section 4. Western blot images and band intensities are represented: panel (**a**)—p-ERK1/2, total-ERK1/2; panel (**b**)—p-p38, total-p38.

To confirm the activation of the ERK and p38 MAPK signaling cascades following AESIS-1 treatment, HDF cells were incubated with AESIS-1 for the indicated durations and analyzed with Western blotting. Figure 6 shows that AESIS-1 treatment induced the phosphorylation of ERK1/2 and p38, which was observed 1 to 5 min into treatment, peaking at 5 min and persisting for 1 h.

Collectively, AESIS-1 increased fibroblast migration and CXCR2 expression through the activation of the ERK and p38 MAPK signaling pathways.

## 3. Discussion

Impaired wound healing affects millions of people worldwide and is associated with significant health and financial burdens, especially for patients with diabetes mellitus and autoimmune disorders such as rheumatoid arthritis [3,36]. The current study shows that the bioactive antirheumatic peptide material AESIS-1 not only accelerates wound closure in an in vivo delayed wound-repair model, but also increases the migration activities of human dermal fibroblasts (Figure 1 and Figure 2). These results mean that AESIS-1 peptides can be used for wound healing, just as DMARDs are used to promote wound healing.

Fibroblasts are the main cells responsible for the decomposition of the fibrin clot; the production of ECMs, including collagen; and wound contraction [12,37]. To initiate the diverse functions of fibroblasts, appropriate and rapid migration into damaged tissue and subsequent proliferation are important determinants of the wound-healing process [12,38]. Figure 2 shows that AESIS-1 treatment increased cell migration in HDF cells, as observed in the transwell migration assay. However, an effect on the proliferation or morphological changes in HDF cells was not observed with AESIS-1 treatment. Even though fibroblast proliferation is an important part of wound healing, the uncontrolled, excessive proliferation of fibroblasts with the associated ECM production can trigger scar formation, such as hypertrophic scarring (HS) and keloids (KDs) [39]. Furthermore, a recent study found that peptide modulators of cell migration have significant applicability to wound healing and tissue regeneration (second largest field, 21%), for example, by inducing fibroblast migration [40]. Applying the AESIS-1 peptide to fibroblast migration acceleration is thus considered a promising approach for enhancing wound repair.

Diverse chemokine receptor expression on infiltrating immune cells and resident cells is involved in the response of various chemokine expressions in damaged tissues to wound repair [16,35]. For example, the expression of CXCR2 in neutrophils, keratinocytes, and endothelial cells is reported elsewhere in the cutaneous wound-healing process [18,35,41,42]. However, the upregulation of CXCR2 on fibroblasts in the wound-healing process has not been sufficiently studied. Here, the treatment of AESIS-1 increased various chemokine receptors, including CCR1, CCR3, CCR7, CCR9, CXCR1, and CXCR2, in HDF cells. Of these increased chemokine receptor expressions, CXCR2 was the most significantly upregulated by AESIS-1 (Figure 3). The upregulation of CXCR2 on fibroblasts induces increased chemotactic activities with CXCL8, which is a well-known counterpart ligand of CXCR2 (Figure 4). Devalaraja et al. reported that CXCR2 knockout mice displayed a delayed wound-healing phenotype in vivo; this reflects the importance of CXCR2 in wound healing. In addition, keratinocytes were considered major sources of CXCR2 expression in their study [17]. Studying the upregulation of CXCR2 and its subsequent functional modification by AESIS-1 in other resident cells, including keratinocytes and endothelial cells, remains open for future studies. CXCR2 is also known to be a major angiogenic chemokine receptor, and interaction with its ligand, CXCL1-8, induces angiogenesis in endothelial cells and models of corneal neovascularization [42,43,44]. Furthermore, blood vessel regeneration is known to be essential for the treatment of the full-thickness wound demonstrated in this study [45,46]. Therefore, the effects of AESIS-1 on angiogenesis should also be studied further.

MAPKs, including ERK, p38, and JNK, are all known to be involved in regulating cell migration, but the regulating mechanisms of each MAPK are distinct [22]. Moreover, the MAPK signaling cascade not only participates in the migration of cells, including cardiac fibroblasts [47], but also in the expression of CXCR1 and CXCR2 in human granulocytes [48]. In this study, AESIS-1 was found to directly activate the ERK and p38 signaling pathways (Figure 6). In addition, inhibitors of the ERK and p38 signaling cascades reduced the migration against CXCL8 and the expression of CXCR2 in HDF cells (Figure 5). The major signaling cascade among MAPKs induced by AESIS-1 treatment for fibroblast migration and CXCR2 expression seems to be the p38 MAPK signaling pathway in our results. Similar results were reported in previous research, which found that p38 MAPK signaling is mainly involved in cell spreading and wound closure by fibroblasts [49].

Although this study identified the potential of AESIS-1 to promote wound healing and presented an action mechanism targeting fibroblasts, it still has limitations. Identifying binding proteins or the receptors of bioactive peptides is challenging because it requires essential research to understand their mode of action at the molecular level [50,51]. Further research should explore the binding proteins or receptors of the AESIS-1 peptide and its distribution in the fibroblasts to understand the exact mode of action. In addition, peptides with antimicrobial properties, including LL-37, have the potential to promote wound healing [52,53,54,55,56]. Thus, it is also worth studying whether the bioactive peptide AESIS-1 has antimicrobial properties based on its therapeutic potential in wound healing, as proven in this study.

In summary, the bioactive peptide AESIS-1 could be a promising candidate for improving wound healing by targeting fibroblast migration enhancement through CXCR2 upregulation.

## 4. Materials and Methods

### 4.1. Peptide Synthesis

The method for AESIS-1 peptide (19 amino acids, MSLPSPRDGRTDGRTDCTR) synthesis was described previously [33]. The purity of the synthetic peptide (>95%) was analyzed using high-performance liquid chromatography (HPLC), and the molecular weight of the synthetic peptide (M.W. 2121.4 g/mol) was determined with liquid chromatography–mass spectrometry (LC-MS).

### 4.2. Cell Culture and Cell Viability

Normal adult human dermal fibroblasts (HDFs) were obtained from ATCC (PCS-201-012, ATCC, Manassas, VA, USA) and cultured in Dulbecco’s modified Eagle’s medium (DMEM)/high glucose (Thermo Fisher Scientific, Waltham MA, USA) supplemented with 100 U/mL penicillin, 100 μg/mL streptomycin, and 10% fetal bovine serum (FBS, Thermo Fisher Scientific, Waltham MA, USA). The cells were frozen in liquid vapor nitrogen at −130 °C until use. Upon thawing, the cells were grown and maintained in a 5% CO_2_ incubator at 37 °C. Cell viability after 24 h of AESIS-1 treatment was measured with a Trypan blue exclusion assay [57]. The HDF cells (2 × 10^5^ cells/6-well plate) were treated with AESIS-1 (1, 10, 100, 1000, 10,000 ng/mL) in serum-free media, and the unstained viable cell number was counted using a hemacytometer after 24 h of incubation.

### 4.3. Wound-Healing Model in Diabetic Mice

A chronic diabetic wound-healing model was induced in mice in vivo, as previously described [58,59]. Briefly, seven-week-old BALB/c-nude mice were purchased from OrientBio (OrientBio Inc., Kyunggido, Republic of Korea) and maintained for one week before starting experimentation. Diabetes was induced with a single intraperitoneal (i.p.) injection of STZ (180 mg/kg B.W. in 0.01 M citrate buffer pH 4.5) into the mice. To confirm the mice were diabetic, mouse tail–vein blood was verified to have a blood sugar concentration higher than 300 mg/dL. After anesthetizing the mice with ketamine (80 mg/kg B.W.) and xylazine (10 mg/kg B.W.), a 6 mm-diameter round punch was used to extract the skin from the back of the animal, causing wounds. Phosphate-buffered saline (PBS) and AESIS-1 peptide (0.5 μg/wound) were topically applied to the wound area five times at 24 h intervals. The wound area was photographed and measured using ImageJ software V 1.8.0 (National Institutes of Health, Bethesda, MD, USA) for 12 days.

### 4.4. Transwell Migration Assay

The fibroblast migration activities were measured with a transwell migration assay, as previously described [60,61]. A transwell chamber (Costar, Boston, MA, USA) with a 6.5 mm diameter and an 8.0 µm pore polycarbonate filter was used in this study. Briefly, 2 μg/mL of mitomycin-C-treated HDF cells (7 × 10^4^ cells) was seeded with serum-free media in the upper chamber, and 1% FBS-supplemented media was added to the lower chamber, followed by 12 h of incubation. The indicated concentrations of AESIS-1 were added to the lower chamber or pre-treated on HDF cells. After the incubation for cell migration, non-migrated cells were removed from the upper compartment of the transwell inserts and fixed with methanol. The lower compartment of the transwell insert was stained with 0.5% crystal violet. For the visualization of cell migration, the lower compartment of the transwell insert was photographed using an IX73 microscope (Olympus Corporation, Tokyo, Japan) and imaging systems. Then, crystal violet dye was eluted with 10% acetic acid, and the absorbance was read using a SpectraMax PLUS Plate Reader (Molecular Devices, San Jose, CA, USA) at 570 nm.

In the transwell migration assay using anti-CXCR2 neutralizing antibody, HDF cells (1.5 × 10^6^ cells/150 mm dish) were pre-treated with 4 μg/mL of human CXCR2/IL-8 RB antibody (cat no. MAB331, Clone#48311, R&D Systems, Minneapolis, MN, USA) for 1 h, and then 20 ng/mL AESIS-1 was applied for 24 h.

### 4.5. Real-Time PCR

The methods for the real-time PCR analysis were described previously [61,62]. A 7900 real-time PCR system (Applied Biosystems, Foster City, CA, USA) with HotStart-IT SYBR-Green qPCR Master Mix (Thermo Fisher Scientific, Waltham MA, USA) was used in this study. The primers used are shown in Table 1. The cycling profile for real-time PCR (50 cycles) was as follows: 95 °C for 10 min, 95 °C for 15 s, and 60 °C for 60 s. The comparative threshold cycle (Ct) method (i.e., 2^−ΔΔCt^) was used to calculate fold amplification.

### 4.6. Flow Cytometry

HDF cells (3 × 10^5^ cells/60 mm dish) were seeded and treated with 20 ng/mL of AESIS-1 peptide for the indicated incubation durations. After incubation, cell surface CXCR2 was stained with 10 μL of FITC-conjugated anti-human CXCR2 antibodies (R&D Systems, Minneapolis, MN, USA) in 100 μL of FACS buffer for 1 h on ice. The labeled cells were then measured and analyzed using FACSCalibur and CellQuest Pro 6.0 software (BD Biosciences, Franklin Lakes, NJ, USA).

### 4.7. Western Blot Analysis

The HDF cells (3 × 10^5^ cells/60 mm dish) were seeded with serum-free media, AESIS-1 peptide was applied for the indicated durations (0, 1, 5, 10, 30, 45, 60 min), and the cells were lysed with RIPA buffer (Cell Signaling Technology, Danvers, MA, USA). The proteins were separated through 8% sodium dodecyl sulfate–polyacrylamide gel electrophoresis (SDS-PAGE) and transferred onto a polyvinylidene difluoride (PVDF) membrane (Bio-Rad, Hercules, CA, USA). After incubation with 5% BSA in PBS blocking solution for 1 h, the target proteins were probed with the following primary antibodies: phospho-ERK, total-ERK, phospho-p38, and total-p38 (Cell Signaling Technology, Danvers, MA, USA). Proteins were visualized using ECL Plus reagent (GE Healthcare, Chicago, IL, USA). The protein band images were quantified with ImageJ software V 1.8.0 (National Institutes of Health, Bethesda, MD, USA).

### 4.8. Statistical Analysis

GraphPad Prism 10 (GraphPad Software, La Jolla, CA, USA) was used for the statistical analyses. Considering the experimental conditions, this study used a Student’s *t*-test, one-way and two-way analysis of variance (ANOVA) with Turkey’s or Dunnett’s multiple comparisons test. A *p*-value ≤ 0.05 was considered statistically significant. The data were expressed as means ± SD.

## 5. Conclusions

In conclusion, this study provided evidence that the rheumatoid arthritis therapeutic bioactive peptide AESIS-1 presents wound-healing activities in a diabetic in vivo model. In particular, AESIS-1 increases the movement and chemotactic activities of fibroblasts by increasing CXCR2 expression. Our findings suggest that AESIS-1 is a therapeutic agent for wound-repair acceleration through the promotion of fibroblast migration.

## Figures and Tables

**Table 1:** Primer sequences for human chemokine receptor genes.

Gene	Title 2	Title 3
*GAPDH*	5′-ATCACCATCTTCCAGGAGCGA-3′	5′-TTCTCCATGGTGGTGAAGACG-3′
*CCR1*	5′-ACCATAGGAGGCCAACCCAAAATA-3′	5′-TCCATGCTGTGCCAAGAGTCA-3′
*CCR2*	5′-CTCTCCCATTGTGGGCTCACTCTG-3′	5′-GCAAACACAGCATGGACAATAGCC-3′
*CCR3*	5′-TTTGTCATCATGGCGGTGTTTTTC-3′	5′-GGTTCATGCAGCAGTGGGAGTAG-3′
*CCR4*	5′-GAGAAGAAGAACAAGGCGGTGAAGA-3′	5′-GGATTAAGGCAGCAGTGAACAAAAG-3′
*CCR6*	5′-CTGCCTGAACCCTGTGCTCTACG-3′	5′-TTATCTGCGGTCTCACTGGTCTGC-3′
*CCR7*	5′-GCCGAGACCACCACCACCTT-3′	5′-AGTCATTGCATCTGCTCCCTATCC-3′
*CCR9*	5′-TATACAGCCAAATCAAGGAGGAATC-3′	5′-CATGACCACGAAGGGAAGGAAG-3′
*CCR10*	5′-GGGCTGGAGTCTGGGAAGTGC-3′	5′-ACGATGACGGAGACCAAGTGTGC-3′
*CXCR1*	5′-CTGAGCCCCAAGTGGAACGAGACA-3′	5′-GCACGGAACAGAAGCTTTATTAGGA-3′
*CXCR2*	5′-CAATGAATGAATGAATGGCTAAG-3′	5′-AAAGTTTTCAAGGTTCGTCCGTGTT-3′
*CXCR4*	5′-AATCTTCCTGCCCACCATCTACTCC-3′	5′-GCGGTCACAGATATATCTGTCATCTGCC-3′
*CXCR5*	5′-TCCCCTCCTCACTCCCTTCCCATAA-3′	5′-CCTGCGGTTCCATCTGAGTGACATC-3′
*CXCR6*	5′-TTGTTTATAGCTTGCGCATTCTCAT-3′	5′-ATCCCCCTTGGTTTCAGCATTCTT-3′

## Data Availability

Data are contained within the article.
